# CCL28: A Promising Biomarker for Assessing Salivary Gland Functionality and Maintaining Healthy Oral Environments

**DOI:** 10.3390/biology13030147

**Published:** 2024-02-27

**Authors:** Yuichiro Kaibori, Saho Tamoto, Sayoko Okuda, Kazuhiko Matsuo, Takashi Nakayama, Daisuke Nagakubo

**Affiliations:** 1 Division of Health and Hygienic Sciences, Faculty of Pharmaceutical Sciences, Himeji Dokkyo University, 7-2-1 Kamiohno, Himeji 670-8524, Hyogo, Japan; yuichiro.kaibori@setsunan.ac.jp (Y.K.);; 2 Laboratory of Analytics for Biomolecules, Faculty of Pharmaceutical Science, Setsunan University, 45-1 Nagaotoge-cho, Hirakata-shi 573-0101, Osaka, Japan; 3 Division of Chemotherapy, Kindai University Faculty of Pharmacy, 3-4-1 Kowakae, Higashi-Osaka 577-8502, Osaka, Japan; matsuo@phar.kindai.ac.jp (K.M.); nakayama@phar.kindai.ac.jp (T.N.)

**Keywords:** salivary gland, biomarker, stem cell, CCL28

## Abstract

**Simple Summary:**

The oral cavity is in direct contact with the outside world. Therefore, the oral cavity becomes a route for external pathogens to enter the host through the air or food. However, saliva supplied from the salivary glands into the oral cavity contains various substances, such as mucus and antibacterial substances, and has an essential function as one of the host’s defense mechanisms. CCL28 is a chemokine expressed in mucosal tissues, such as the colon, and is also abundantly expressed in the salivary glands. However, the effect of CCL28 on saliva production has not yet been apparent. In this study, we investigated the effect of CCL28 on saliva production using CCL28-deficient mice. CCL28-deficient mice have decreased production of mucus, which makes up saliva. In addition, in the submandibular gland, which is one of the salivary glands, CCL28-deficient mice showed decreased expression of cytokeratin 18, a marker molecule for salivary gland ductal cells, reduced expression of ductal cell F-actin, which indicates cell polarity, and a decrease in the number of ducts. Furthermore, CCL28-deficient mice showed decreased expression of assumed marker molecules of salivary gland stem cells. These results suggest the potential of CCL28 as a functional biomarker for salivary glands.

**Abstract:**

The oral cavity serves as the primary path through which substances from the outside world enter our body. Therefore, it functions as a critical component of host defense. Saliva is essential for maintaining a stable oral environment by catching harmful agents, including pathogens, allergens, and chemicals, in the air or food. CCL28, highly expressed in mucosal tissues, such as the colon and salivary glands, is a chemokine that attracts CCR10/CCR3 expressing cells. However, the role of CCL28 in salivary gland formation remains unclear. In this study, we investigated the salivary gland structure in CCL28-deficient mice. Histological analysis showed decreased staining intensity of Alcian blue, which detects acidic mucous, reduced expression of MUC2, and higher infiltration of gram-positive bacteria in the salivary glands of CCL28-deficient mice. In addition, CCL28-deficient mice contained ectopically MUC2-expressed cells in the ducts and reduced the expression of cytokeratin 18, a marker for ductal cells, within the submandibular glands, resulting in decreased duct numbers. Additionally, the submandibular glands of CCL28-deficient mice showed reduced expression of several stem cell markers. These results suggest that CCL28 regulates saliva production via proper differentiation of salivary gland stem cells and could be a valuable biomarker of salivary gland function.

## 1. Introduction

The importance of the oral cavity covered with mucous membranes is definite from the perspective of host defense [1]. The oral cavity is directly open to the outside world. Thus, it can be an entry site for pathogens, such as microorganisms present in the air or attached to food, to enter the body. Therefore, the adequate maintenance of oral function reduces the risk of infection through the respiratory tract and gastrointestinal mucosa [2,3,4]. Certain bacteria cause oral diseases. *Streptococcus mutans*, which is the cause of caries, and *Porphyromonas gingivalis*, *Tannerella forsythia*, and *Treponema denticola*, which are the causes of periodontal disease, are particularly well known. Furthermore, oral microbiomes consisting of microorganisms, including bacteria, archaea, fungi, and viruses in the oral cavity, have attracted attention. Loss of saliva production, as observed in older people and patients with Sjögren’s syndrome (SS), an autoimmune disease characterized by xerostomia and dry eyes, causes oral dysbiosis, a state in which the composition and balance of the oral microbiome are disturbed [5]. It is becoming clear that oral dysbiosis can cause systemic diseases, including chronic diseases, such as heart disease and diabetes [4,6,7]. Thus, changes in the microbial environment formed in the oral cavity are critical because they seriously affect the health of the human host.

Saliva and various salivary factors influence the oral cavity environment [8]. Saliva is a cleaning fluid in the oral cavity, contributes to keeping the oral environment clean by washing away food debris in the oral cavity, and protects the teeth and oral mucosa from physical and chemical damage [9]. In addition, saliva facilitates chewing and swallowing [9,10]. In older people, a decrease in saliva due to aging causes difficulty in swallowing, which is one of the causes of aspiration pneumonia. Other functions of saliva include the digestion of food, maintenance of oral pH, repair and regeneration of oral tissues, and detection of taste [9]. Therefore, saliva is essential for maintaining oral cavity functionality.

Saliva contains numerous proteins that are immunologically important for the host defense against pathogens. A typical example is the antibacterial protein, lysozyme, which breaks down the cell walls of pathogenic bacteria and prevents their growth [11]. Other proteins, such as agglutinins, lactoferrin, lactoperoxidase [8], and the salivary extracellular chaperone HSP70/HSPA protein family [12], also have antibacterial effects. Furthermore, secretory IgA, an immunoglobulin in the saliva, also plays a crucial role in coagulating bacteria and preventing them from adhering to the oral mucosa [13]. In addition, the glycoprotein, mucin, in the oral cavity prevents bacteria from adhering to the oral mucosa. Particularly, MUC5B and MUC7, which are abundantly secreted mucins in saliva, are highly glycated and play a marked role [12]. The salivary glands are responsible for producing and secreting saliva and for secreting the factors involved in host defense, such as IgA and mucins like MUC5B and MUC7 [12,14]. There are three major salivary glands in the oral cavity, the submandibular gland (SMG), and sublingual gland (SLG), and parotid gland (PG), as well as numerous minor salivary glands within the oral submucosa [9].

Salivary glands, primarily receiving innervation from the autonomic nervous system, produce saliva secreted into the oral cavity in mammals such as humans and mice. Precisely, sympathetic nerves and parasympathetic nerves regulate the production of mucous saliva and serous saliva, respectively, in the salivary gland [15]. Stimulation of sympathetic nerves by isoproterenol treatment, an agonist of β-adrenergic receptors, contributes to the regeneration of salivary tissues via acinar cell proliferation and hypertrophy, accompanied by the promotion of the mucous saliva production [16,17,18]. Meanwhile, stimulation of parasympathetic nerves promotes serous saliva production via the activation of M3 or M1 muscarinic receptors [15]. In addition, parasympathetic denervation causes salivary gland atrophy in humans and rodents, and reinnervation of parasympathetic nerves reversed the atrophy, indicating that stimulation of parasympathetic nerves is essential for maintaining salivary glands [15,19]. Regarding the regulation of the salivary water content, AQP5, a member of the water channels that transports water into the lumen [20], has been demonstrated to play an essential role in saliva production since AQP5-deficient mice showed decreased saliva production [21]. It has been revealed that the parasympathetic nervous system regulates the expression of AQP5 in salivary gland acinar cells during development [22]. However, the detailed regulatory mechanism of AQP5 expression in adults is unclear.

CCL28 is a chemokine abundantly expressed in mucosal tissues, such as the mammary glands, trachea, and colon, and contributes to the migration of IgA^+^ antibody-secreting cells (ASC) that express CCR10 [23]. Salivary glands, including SMG, SLG, and PG, also express CCL28, which has potent and broad-spectrum antimicrobial activity against *Candida albicans* and gram-negative and gram-positive bacteria [24]. We previously reported that the colons of CCL28-deficient mice possess an altered microbiome that exacerbates dextran sulfate sodium-induced colitis [25]. Thus, CCL28 could be considered an essential factor for host defense in mucosal tissues, such as the gastrointestinal tract. However, its role in salivary gland function and its relation with the saliva produced in the oral cavity remain unclear.

In this study, we attempted to elucidate other functional consequences of CCL28 in the oral cavity. In the salivary glands of CCL28-deficient mice, we found reduced mucus and reduced expression of molecules, such as AQP5 and secreted mucin MUC2. Furthermore, we observed microstructural alterations in the SMG, as highlighted by abnormalities in the organization of ductal cells, in CCL28-deficient mice. We also suggest that this change may be related to differentiation or functional abnormalities of salivary gland stem cells in the SMG. Abnormal SMG in CCL28-deficient mice results in tissue infiltration by gram-positive bacteria. These findings suggest that CCL28 plays a vital role in creating a healthy oral environment, including the microbiome, by influencing salivary gland differentiation and organization, and producing functional saliva. Therefore, we propose that CCL28 is a valuable biomarker for the regulation of saliva production.

## 2. Materials and Methods

### 2.1. Mice

CCL28-deficient (CCL28^−/−^) mice on a C57BL/6 background were generated as previously described [25]. We crossed them with C57BL/6 mice, which were purchased from CLEA Japan Inc. (Tokyo, Japan), to produce CCL28 heterozygous (CCL28^+/−^) mice. The heterozygous mice were then interbred to generate both CCL28-deficient and wild-type (WT) (CCL28^+/+^) mice, which were used at 8–10 weeks of age for this study. All murine strains were maintained under conventional conditions.

### 2.2. Histology and Immunofluorescence Staining

Histological analyses and immunofluorescence staining were performed as previously described [25,26,27,28] using frozen or paraffin sections. In brief, for frozen sections, SMGs and SLGs were obtained, fixed with 1% paraformaldehyde for 24 h at 4 °C, incubated in 20% sucrose in PBS(-) for 24 h at 4 °C, and embedded in an OCT compound (Sakura Finetek Japan, Tokyo, Japan). For paraffin sections, SMGs and SLGs were fixed, dehydrated, and then embedded in paraffin. Sections were prepared with a thickness of 8 μm. For Hematoxylin and Eosin (H&E) staining, sections were rehydrated, stained with H&E, and mounted with malinol (Muto Pure Chemicals, Tokyo, Japan). For Alcian blue (AB) staining, sections were rehydrated, incubated in AB Solution (pH 2.5, FUJIFILM Wako, Tokyo, Japan) for 30 min, rinsed with 3% acetic acid, and washed in water. After incubation in Kernechtrot Stain Solution (Muto Pure Chemicals) for 1 min for counterstaining, sections were mounted with malinol. For Periodic acid-Schiff (PAS) staining, the sections were incubated in 0.5% Periodic acid solution (Muto Pure Chemicals) for 10 min, followed by staining with Schiff’s reagent (Muto Pure Chemicals) for 30 min. They were then rinsed three times for 5 min each using a sulfite solution (NaHSO_3_/HCl solution, Muto Pure Chemicals) and finally stained with a hematoxylin solution for 15 min. For Gram staining, sections were stained with a Gram staining kit for tissues (Gram-Hucker’s solution I–III) (Muto Pure Chemicals), following the manufacturer’s instructions. Images were obtained using a BX53 microscope (EVIDENT, Tokyo, Japan) and analyzed. For immunofluorescence staining, sections were blocked with PBS containing 2% BSA and 1% donkey serum and incubated with primary and secondary antibodies. Fluorescent images were obtained using a confocal laser scanning microscope LSM510 (Carl Zeiss, Oberkochen, Germany). The intensity of AB staining was measured by extracting and analyzing the blue color using ImageJ software (version 1.53t). Similarly, the fluorescence intensities of Ulex europaeus agglutinin-1 (UEA-1) and MUC2 were measured using the ImageJ software.

### 2.3. RT-PCR

RT-PCR was performed as previously described [29]. Briefly, SMGs obtained from WT and CCL28-deficient mice were lysed in TRIzol reagent (Thermo Fisher Scientific, Waltham, MA, USA), and cDNA was prepared by RT using the ReverTra Ace qPCR RT Master Mix (Toyobo, Osaka, Japan). Each gene was amplified using KOD FX Neo DNA polymerase (Toyobo). The conditions of PCR were 98 °C for 30 s (denaturation), 60 °C for 30 s (annealing), and 68 °C for 30 s (extension) for 29–42 cycles. The following primer pairs were used: 5′-AAGCCAGCTCCGGCATCATC-3′ and 5′-GCTGCAGGGGTCTGGGTTGT-3′ for *MUC2*, 5′-TCTACTTCTACTTGCTTTTCCCCTCCTC-3′ and 5′-CGATGGTCTTCTTCCGCTCCTCTC-3′ for *AQP5*, 5′-TGCAGGTCTCTCCACCCAAT-3′ and 5′-CCCAACCTTCTCCCCAGTTC-3′ for *α-Amylase*, 5′-ACTGGGACGACATGGAAAAG-3′ and 5′-AGGCCCCTGCAGGTTTTGAAG-3′ for alpha-smooth muscle actin (*α-SMA*), 5′-AAGGTGAAGCTTGAGGCAGA-3′ and 5′-CTGCACAGTTTGCATGGAGT-3′ for cytokeratin 18 (*CK18*), 5′-TGGTTGTGGTTGTTGTTGTTGTTG-3′ and 5′-GAAGGCTTGTTCCGAAGTGTAGAC-3′ for *c-Kit*, 5′-GCCACGTGCTGGTGTGTCAA-3′ and 5′-CGAGCGGCTCTCCGTTCACT-3′ for *EpCAM*, 5′-AGAGCTCTGTTACAAGGCTGATGTC-3′ and 5′-CAGGTGGTACTTCCTAGATTCCAGC-3′ for *CCR10*, 5′- CAGCCCGCACAATCGTACT-3′ and 5′- ACGTTTTCTCTGCCATTCTTCTTT-3′ for *CCL28*, 5′-TGGGACTCACGCTGGTGCTC-3′ and 5′-GTCACGACCCCGCCTTCTGT-3′ for *CD31*, and 5′-GCCAAGGTCATCCATGACAACTTTGG-3′ and 5′-GCCTGCTTCACCACCTTCTTGATGTC-3′ for *GAPDH*. For the display of the relative expression level of each gene in the graph, the intensity of each PCR band was measured using ImageJ software, and the statistical analysis was performed based on normalizing the measured values of each target gene using the GAPDH values.

### 2.4. Antibodies

The primary antibodies used for immunofluorescence analyses were as follows: Rabbit polyclonal anti-MUC2 (PA5-79702, Invitrogen, Carlsbad, CA, USA) and anti-α-SMA (PA5-87638, Invitrogen); Goat polyclonal anti-CK18 (sc-31700, Santa Cruz Biotechnology, Dallas, TX, USA), anti-mouse CCL28 (AF533, R&D Systems, Minneapolis, MN, USA), and anti-mouse CCR10 (AAM69, Bio-Rad Laboratories, Hercules, CA, USA); Rat monoclonal anti-EpCAM (clone G8.8, BioLegend, San Diego, CA, USA), anti-c-Kit (clone 2B8, BioLegend), and anti-CD31 (clone MEC13.3, BioLegend) antibodies. Alexa Fluor 488- or 555-labeled donkey anti-mouse, donkey anti-rabbit, or goat anti-rat IgG (Invitrogen) antibodies were used as secondary antibodies. DAPI (Dojindo Laboratories, Kumamoto, Japan) was used for DNA staining. Fluorescein isothiocyanate-conjugated UEA-1 (Invitrogen) and phalloidin (Invitrogen) were used for lectin and F-actin staining, respectively.

### 2.5. Statistical Analysis

Student’s *t*-test was performed to analyze the difference between the two groups. *p* values < 0.05 were considered significant.

## 3. Results

### 3.1. Deletion of CCL28 Reduces Acidic Mucin Production in Both SMG and SLG and Promotes Bacterial Invasion

**Figure 1:**
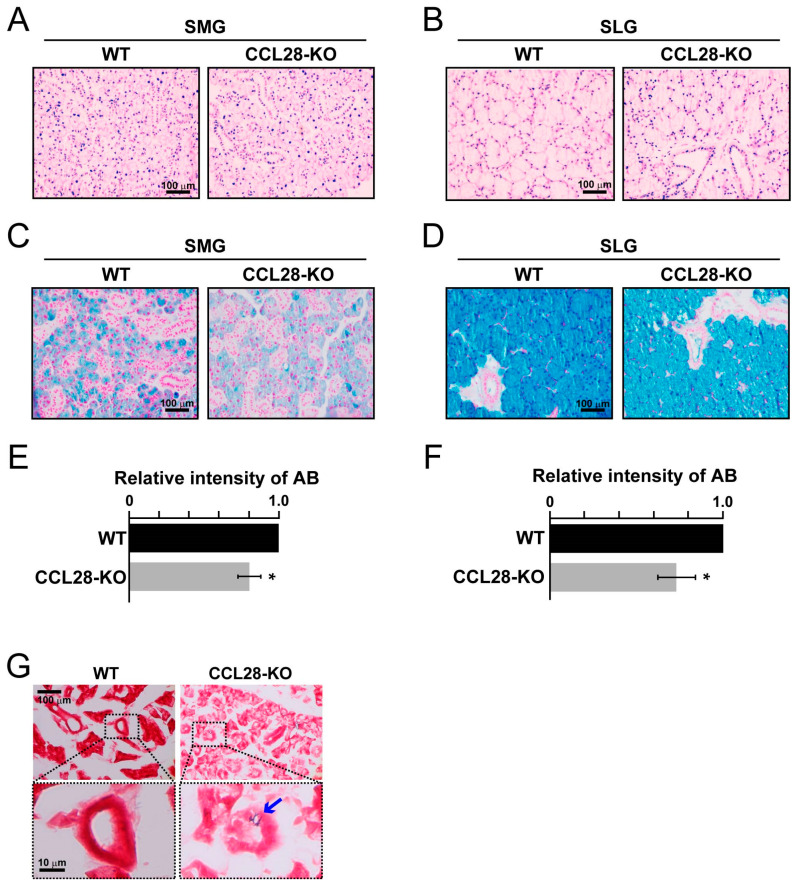
CCL28-deficient mice have reduced acidic mucin production in SMG and SLG, with bacterial invasion occurring in their SMG. (**A**–**F**) SMG and SLG were obtained from WT or CCL28-deficient (CCL28-KO) mice. After preparing the sections, H&E staining (**A**,**B**) and AB staining (**C**,**D**) were performed, respectively. Representative images are shown. Scale bars: 100 μm. Relative intensities of AB staining are shown as the means ± SD of results from three independent experiments. *, *p* < 0.05 (**E**,**F**). (**G**) Sections of the SMG from WT or CCL28-KO mice were stained with Gram-Hucker’s solution. Representative images are shown. Scale bar: 100 μm (upper panel). The lower panel shows an enlarged image of the dotted area in the upper panel. Scale bar: 10 μm (lower panel). The arrow indicates the gram-positive bacteria.

As for the *in vivo* function of CCL28, there have been no reports regarding its role in saliva production, a unique function of the salivary glands. Therefore, we examined the effect of CCL28 on salivary production in CCL28-deficient mice. Initially, we performed H&E staining to check for histological conditions, and then stained acidic mucins in the salivary glands with AB. Acidic mucin is a component of mucus in saliva. H&E-stained images showed no noticeable structural differences in the SMG and SLG of CCL28-deficient mice compared with those of WT mice (Figure 1A,B). Meanwhile, in both the SMG and SLG, the intensities of AB staining were significantly decreased in CCL28-deficient mice than in WT (Figure 1C–F). Furthermore, although we rarely detected gram-positive bacteria in the SMG of WT mice, they were detected at some frequency in the SMG of CCL28-deficient mice (Figure 1G). In contrast, we did not detect gram-positive bacteria in the SLG of either WT or CCL28-deficient mice (Figure S1). These results suggest that CCL28 regulates mucus production in the salivary glands, which may also contribute to the protection against bacterial invasion of the salivary glands, especially the SMG, *in vivo*. Considering the results of Gram staining analysis, we decided to focus on SMG in subsequent experiments.

We also confirmed the expression of CCL28 and its receptor, CCR10, using RT-PCR and immunohistochemistry staining (Figure S2). CCL28 was reliably expressed in the SMG at the mRNA level, as previously reported [24] (Figure S2A). Regarding CCR10, which IgA^+^ ASCs bear in the mucosal tissue, no significant difference was observed in the mRNA expression in the whole SMG between WT and CCL28-deficient mice (Figure S2A,B). As for protein expression, we previously reported that CCL28 was expressed in epithelial cells in the SLG [25]. In the SMG, we similarly confirmed CCL28 expression in epithelial cells, especially ductal cells (Figure S2C). We found that CCR10, like its ligand CCL28, was also expressed in ductal cells (Figure S2D). We previously detected decreased IgA^+^ ASCs in the SLG of CCL28-deficient mice compared with WT mice [25]. However, considering that there was no significant difference in the levels of CCR10 mRNA in the SMG between WT and CCL28-deficient mice (Figure S2A,B), the predominant cells expressing CCR10 in the SMG would be ductal, implying that CCL28 might directly affect ductal cells in the SMG via CCR10.

### 3.2. Deletion of CCL28 Diminishes the Expression of Salivary Gland Markers

**Figure 2:**
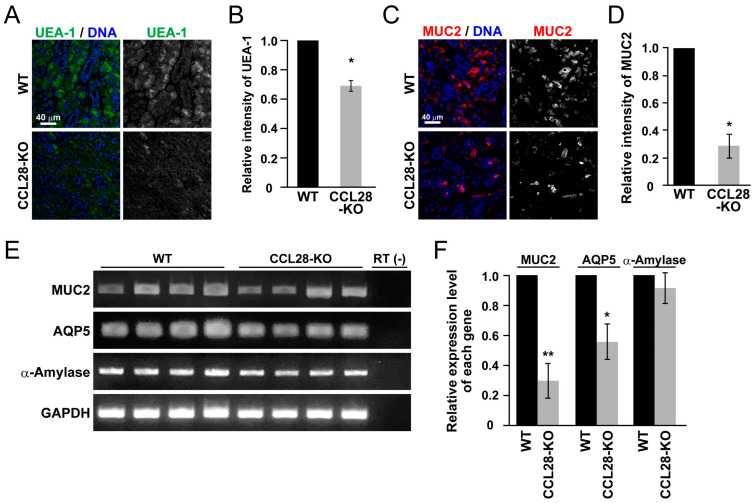
CCL28-deficient mice have altered expression of salivary gland markers in the SMG. (**A**) Sections of SMG from WT or CCL28-KO mice were stained for UEA-1 (green) and DNA (blue). Representative images are shown. Scale bar: 40 μm. The right panel shows the same image as the left panel, with the blue signal of the nuclear stain omitted and only the green signal of UEA-1 shown in white. (**B**) Relative intensities of UEA-1 staining are shown as the means ± SD of results from three independent experiments. *, *p* < 0.05. (**C**) Sections of SMG from WT or CCL28-KO mice were stained for MUC2 (red) and DNA (blue). Representative images are shown. Scale bar: 40 μm. The right panel shows the same image as the left panel, with the blue signal of the nuclear stain omitted and only the red signal of MUC2 shown in white. (**D**) Relative intensities of MUC2 staining are shown as the means ± SD of results from three independent experiments. *, *p* < 0.05. (**E**) Semi-quantitative RT-PCR for mRNA expression of MUC2, AQP5, α-Amylase, and GAPDH was performed using cDNA prepared from the SMG derived from each of the four WT or CCL28-KO mice. (**F**) The relative expression level of each indicated gene, analyzed using the values obtained by measuring the bands in (**E**), is shown as the means ± SD of results from four independent mice. *, *p* < 0.05, **, *p* < 0.01.

Since we observed impaired salivary gland function in CCL28-deficient mice, we examined the expression of several markers to gain insight into the salivary gland conditions in CCL28-deficient mice. First, to further investigate the reduced acidic mucin production in the SMG and SLG of CCL28-deficient mice (Figure 1), we analyzed the expression of lectin and mucin. As shown in Figure 2A,B, we found that the fluorescence intensity of lectin UEA-1, a glycan-binding protein, was significantly decreased in the SMG of CCL28-deficient mice compared with that in WT mice. Subsequently, we investigated MUC2, a secreted gel-forming mucin expressed in the SMG [30], as one of the mucins. Regarding MUC2, we assumed that MUC2 would be necessary from an immune perspective in the SMG because analyses of the mouse colon clarified that probiotics alter MUC2 expression [31], and studies of mouse salivary glands implied that MUC2 might function in the bacterial infection response [32], although the absolute MUC2 expression level is relatively low. As shown in Figure 2C,D, the immunofluorescence intensity of MUC2 was significantly decreased in the SMG of CCL28-deficient mice compared with that in WT mice. To further verify the influence of CCL28 on SMG function, we examined the expression of several genes using RT-PCR. Although there were slight differences in expression between individuals, CCL28-deficient mice showed a significant decrease in the expression of AQP5, similar to the significantly reduced expression of MUC2 (Figure 2E,F). In contrast, the expression of α-Amylase, the most abundant digestive enzyme in saliva for cleavage of large starch molecules and to support food digestion [33], was comparable between WT and CCL28-deficient mice (Figure 2E,F). These results indicate that CCL28 regulates the functions of the salivary glands in mucous and serous saliva generation through AQP5.

### 3.3. Deletion of CCL28 Causes Alterations in the Ductal Structure of SMG

**Figure 3:**
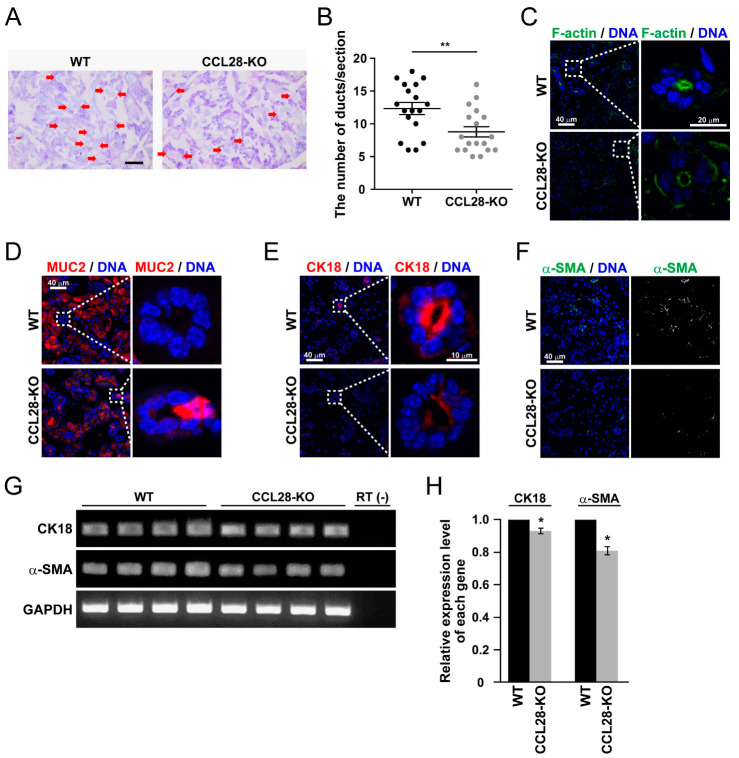
CCL28-deficient mice lead to structurally defective SMG formation. (**A**) Sections of SMG from WT or CCL28-KO mice were stained with PAS reagent. Representative images are shown. Scale bar: 100 μm. The arrows in the image indicate the duct in which the entire structure was recognized. (**B**) The number of ducts in which the entire structure was recognized, per PAS-stained image of SMG sections from WT or CCL28-KO mice, respectively, was counted as the means ± SD of results from three independent experiments. **, *p* < 0.01. (**C**) Sections of the SMG from WT or CCL28-KO mice were stained for Phalloidin (F-actin; green) and DNA (blue). Representative images are shown. Scale bar: 40 μm (left panel). The right panel shows an enlarged image of the dotted area in the left panel. Scale bar: 20 μm (right panel). (**D**) Sections of the SMG from WT or CCL28-KO mice were stained for MUC2 (red) and DNA (blue). Representative images are shown. Scale bar: 40 μm (left panel). The right panel shows an enlarged image of the dotted area in the left panel. Scale bar: 10 μm (right panel). (**E**) Sections of the SMG from WT or CCL28-KO mice were stained for CK18 (red) and DNA (blue). Representative images are shown. Scale bar: 40 μm (left panel). The right panel shows an enlarged image of the dotted area in the left panel. Scale bar: 10 μm (right panel). (**F**) Sections of the SMG from WT or CCL28-KO mice were stained for α-SMA (green) and DNA (blue). Representative images are shown. Scale bar: 40 μm. The right panel shows the same image as the left panel, with the blue signal of the nuclear stain omitted and only the green signal of α-SMA shown in white. (**G**) Semi-quantitative RT-PCR for mRNA expression of CK18, α-SMA, and GAPDH was performed using cDNA prepared from the SMG derived from each of the four WT or CCL28-KO mice. (**H**) The relative expression level of each indicated gene, analyzed using the values obtained by measuring the bands in (**G**), is shown as the means ± SD of results from four independent mice. *, *p* < 0.05.

Although we demonstrated that CCL28 functionally regulates the salivary glands, we did not observe marked tissue abnormalities of the SMG and SLG in CCL28-deficient mice by H&E staining (Figure 1A,B). Therefore, we quantitatively examined the structural characteristics of SMG in more detail. As shown in Figure 3A,B, by PAS staining, which indicates the neutral mucus [34], CCL28-deficient mice exhibited a significant decrease in the number of ducts in the SMG.

Exocrine glands, such as the mammary, salivary, and sweat glands, form a lumen to excrete exocrine fluids like milk, saliva, or sweat [35]. The polarity of the cells comprising the lumen is strictly defined to secrete fluid properly. F-actin typically accumulates in the apical region of the lumen to maintain its polarity via interaction with ZO-1 [36]. However, the fluorescence intensity of F-actin on the apical side of the ducts in the SMG of the CCL28-deficient mice was markedly reduced (Figure 3C). Further analysis of the ducts in the SMG revealed that MUC2 was ectopically expressed in ductal cells of CCL28-deficient mice (Figure 3D). In addition, CK18, which is typically localized in the ductal cells in adult mice [37], and α-SMA, a marker of myoepithelial cells, which supports the secretion of saliva into the oral cavity [38], were decreased in CCL28-deficient mice (Figure 3E,F). We also performed semi-quantitative RT-PCR analyses to confirm the reduction in the protein expression of these genes. The expression of both CK18 and α-SMA were significantly reduced in the SMG of CCL28-deficient mice compared with that in WT mice (Figure 3G,H). These changes in the expression and localization of functional proteins in the SMG caused by the deletion of *CCL28* could lead to the formation of SMG tissues with intrinsic abnormalities. These results suggest that CCL28 contributes to the formation of SMG tissue by inducing cell type-specific gene expression or by allocating individual cells to their proper sites.

### 3.4. CCL28 Regulates the Differentiation of the Stem Cells in the Salivary Gland

**Figure 4:**
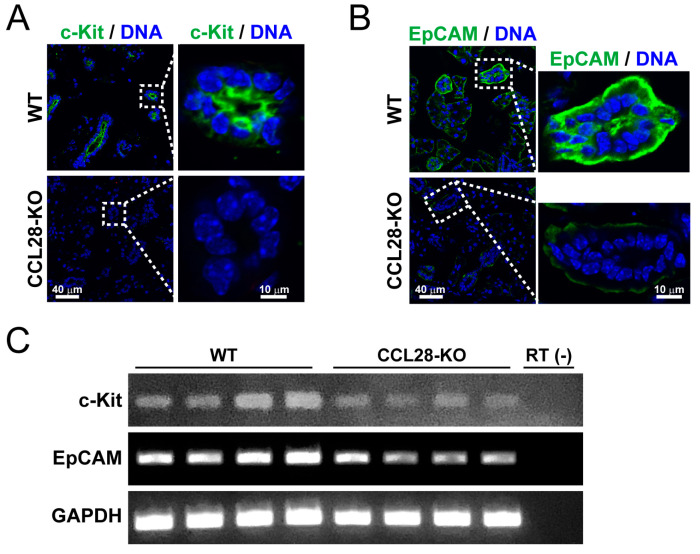
CCL28-deficient mice have reduced expression of stem cell markers in SMG. (**A**) Sections of the SMG from WT or CCL28-KO mice were stained for c-Kit (green) and DNA (blue). Representative images are shown. Scale bar: 40 μm (left panel). The right panel shows an enlarged image of the dotted area in the left panel. Scale bar: 10 μm (right panel). (**B**) Sections of the SMG from WT or CCL28-KO mice were stained for EpCAM (green) and DNA (blue). Representative images are shown. Scale bar: 40 μm (left panel). The right panel shows an enlarged image of the dotted area in the left panel. Scale bar: 10 μm (right panel). (**C**) Semi-quantitative RT-PCR for mRNA expression of c-Kit, EpCAM, and GAPDH was performed using cDNA prepared from the SMG derived from each of the four WT or CCL28-KO mice.

We found that the normal regulation of the expression of marker molecules for SMG components was impaired in CCL28-deficient mice. Therefore, we investigated whether CCL28-deficient mice exhibit defective differentiation of salivary gland stem cells. Although stem cells in the salivary glands have not been definitively identified, c-Kit has been suggested as a potent marker for salivary gland stem cells [39,40,41,42]. In addition, higher EpCAM expression is essential for the long-term expansion and differentiation of salivary gland stem cells [43]. Hence, we analyzed the expression of these markers in the SMG of WT and CCL28-deficient mice. Although both c-Kit and EpCAM were expressed in the ductal cells of WT mice, immunofluorescence staining revealed a considerable reduction in c-Kit- and EpCAM-positive cells in CCL28-deficient mice (Figure 4A,B). By analyzing the mRNA expression of these genes, we also confirmed a reduction in the expression levels of c-Kit and EpCAM mRNA in the SMG of CCL28-deficient mice (Figure 4C), consistent with the immunofluorescence analysis. These results suggested that the decreased number of putative salivary gland stem cells in CCL28-deficient mice generate dysfunctional cells in the SMG.

### 3.5. Angiogenesis for the Organization of the Salivary Gland Will Require CCL28

**Figure 5:**
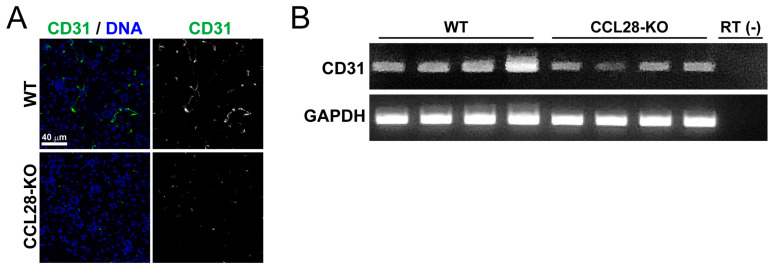
CCL28-deficient mice have reduced expression of CD31, a vascular endothelial marker in SMG. (**A**) Sections of the SMG from WT or CCL28-KO mice were stained for CD31 (green) and DNA (blue). Representative images are shown. Scale bar: 40 μm. The right panel shows the same image as the left panel, with the blue signal of the nuclear stain omitted and only the green signal of CD31 shown in white. (**B**) Semi-quantitative RT-PCR for mRNA expression of CD31 and GAPDH was performed using cDNA prepared from the SMG derived from each of the four WT or CCL28-KO mice.

In tumor tissues under hypoxic environments, CCR3 expressed in vascular endothelial cells [44], and CCR10 expressed in T_reg_ cells [45], both receptors for CCL28, have been reported to contribute to promoting the angiogenesis process through hypoxia-induced CCL28. Therefore, we investigated whether CCL28 promoted angiogenesis in SMG. As shown in Figure 5A, the number of CD31-positive cells, a marker of vascular endothelial cells [46], was decreased in CCL28-deficient mice. Semi-quantitative RT-PCR analysis also indicated a consistent reduction in CD31 mRNA expression in the SMG of CCL28-deficient mice (Figure 5B).

Collectively, our study revealed that CCL28 is essential for the formation of normal functional salivary gland tissues.

## 4. Discussion

In this study, we discovered that CCL28 is involved in acquiring SMG functionality via SMG organization. Studies on the relation between the salivary glands and CCL28, which is abundantly expressed in mucosal tissues, have included analyses of patients with SS. Primary SS (pSS) is an autoimmune disease characterized by dry tissue, such as the oral cavity and eyes, due to damage in the exocrine glands caused by immune cells. Blokland et al. summarized various chemokines involved in immune cell infiltration, leading to tissue inflammation in pSS [47]. Hernandez-Molina et al. reported that CCL28 was significantly reduced in the saliva of patients with pSS compared with that in healthy controls [48]. Meanwhile, patients with SS have virtually no saliva, making saliva collection difficult. Yu et al. reported that serum CCL28 levels were decreased in patients with both primary and secondary SS [49]. These results indicate that CCL28 can be used as a biomarker for diagnosing SS. Furthermore, these insights raise the possibility that functional CCL28 could influence the production and functionality of saliva in the salivary glands, even under physiological conditions.

We analyzed mucus production in the SMG and SLG of CCL28-deficient mice and found that these salivary glands showed reduced mucus production (Figure 1C–F). In addition, CCL28-deficient mice also showed significantly decreased expression of AQP5 in the SMG (Figure 2F), suggesting that CCL28 may influence the serous production function of the SMG. Consequently, using CCL28-deficient mice, we provided evidence in this study for the possibility assumed from the analyses of patients with SS. In contrast, a study by Yu et al. showed significantly increased CCL28 mRNA expression in the PG of the xerostomia group compared with that in the healthy control group, whereas CCL28 mRNA expression was significantly decreased in the PG of the patient with SS group compared with that in the healthy control group [49], implying that the effect of CCL28 on the PG, primarily composed of serous glands, under physiological conditions, remains unknown. Furthermore, there are some differences between humans and mice in the structure of salivary glands. For example, PG is the largest salivary gland in humans, whereas the size of PG and SMG is comparable in mice [19]. In both SMG and PG, which contain serous acinar cells, the production of serous saliva is regulated by parasympathetic nerves [15]. However, there have been no reports on the regulation of the autonomic nervous system by CCL28. Therefore, the significance of CCL28 expression in human salivary glands will require further analysis using human clinical samples, even though in our present study, we used an experimental mouse system to demonstrate the potential of CCL28 as a biomarker for evaluating salivary gland functionality.

Mucin, which constitutes the mucus contained in saliva, is an essential barrier that protects the host’s oral mucosa from microorganisms, such as bacteria, and prevents them from invading tissues [50]. In the present study, we demonstrated the infiltration of gram-positive bacteria into the SMG of CCL28-deficient mice (Figure 1G). However, since CCL28 has broad antimicrobial effects against bacteria [24] and chemoattractant activity on CCR10-positive IgA^+^ ASC [23], the antimicrobial activity of CCL28 itself and the immunological efficacy of secretory IgA would also act against bacteria simultaneously. Therefore, a combination of these multiple direct and indirect actions of CCL28 may provide greater protection against tissue invasion by bacteria. In contrast, bacterial invasion within the SLG did not occur, even in CCL28-deficient mice (Figure S1), possibly because of the large amount of mucus compared with that in the SMG.

The present study revealed microstructural changes in the SMG of CCL28-deficient mice (Figure 3A,B). Tissue stem cells are responsible for continuous regeneration or, when needed, for constant self-renewal and differentiation in various tissues. The low cellular turnover of salivary glands, including stem cells, is a disadvantage for the accurate identification of salivary gland stem cells, which has not yet been achieved [51]. Nevertheless, because salivary gland reconstitution occurs even after acute disruptions, such as irradiation of the head and neck, stem cells are thought to exist in the salivary glands [52,53]. Irradiation analyses identified several candidate molecules that are assumed to be stem cell markers, such as c-Kit, EpCAM, and Lgr5, in the salivary gland [39,40,41,42,43,54,55]. As a chemokine involved in the regulation of tissue stem cells, the previous analyses of CXCL12 have been impressive. CXCL12, through its receptor CXCR4, is responsible for the maintenance and differentiation of tissue stem cells, such as bone marrow hematopoietic stem cells, primordial germ cells, and neural stem cells, in the stem cell niche of the tissue microenvironment [56,57,58]. This function may be mainly based on the chemoattractant activity of CXCL12 toward the stem cell niche. F-actin staining revealed abnormal ductal polarity in the SMG of CCL28-deficient mice (Figure 3C). In addition, we revealed the incomplete organization of duct cells and structural fragility in the SMG of CCL28-deficient mice by the ectopic localization of MUC2-positive cells in ducts, decreased expression of CK18 in ductal cells, and reduced frequencies of α-SMA^+^ myoepithelial cells promoting saliva production, as niche (Figure 3D–F). These results, which are reflected by reduced ductal numbers and CD31^+^ vasculature (Figure 3A,B and Figure 5), suggest that the functional abnormality in saliva production might be due to the abnormal microstructure of the SMG. As a plausible reason, in the SMG of CCL28-deficient mice, the expression of molecules considered to be markers of salivary gland tissue stem cells, such as c-Kit and EpCAM, was reduced (Figure 4), implicating that the correct differentiation mechanism might not be induced. It is possible that CCL28, similar to CXCL12, is involved in constructing a stem cell niche in the salivary gland, driving the recruitment of salivary gland stem cells or inducing differentiation through genetic induction in salivary gland stem cells.

Previous analyses using salivary gland excretory duct ligation models in rats and mice have shown that ligation causes acinar cell loss and atrophy, decreasing AQP5 expression but inducing ductal cell proliferation [59,60]. In the present study, CCL28-deficient SMG showed decreased AQP5 expression (Figure 2E,F) and reduced number of duct structures (Figure 3A,B). Accordingly, this could be considered a phenotype that is different from that of the ligated animal model. Therefore, research on salivary gland regeneration using CCL28-deficient mice might reveal a molecular mechanism distinct from the regeneration process after ligation in animal models of excretory duct ligation, and CCL28-deficient mice could be helpful for salivary gland regenerative medicine research.

Based on the results of the above analyses, we suggest that CCL28 is a potential biomarker that can indicate the functionality of salivary glands. Several molecules, such as cortisol and α-amylase, have been recognized and used as salivary biomarkers that increase due to psychological stress [61,62]. In addition, many other types of biomarkers related to diseases, such as chronic diseases and tumors, have been reported, including nucleic acids, hormones, and enzymes [63]. Thus, saliva has many advantages as a non-invasive body fluid. Our discovery is novel in that chemokines can be used as biomarkers and provides a new concept in that CCL28 can be a candidate indicator for evaluating the functionality of the salivary glands that regulate saliva production. Nevertheless, α-amylase has already been proposed as a similar concept as an indicator to assess salivary gland function in patients with oral cancer who have undergone radiation therapy [64].

More detailed analyses of changes in CCL28 expression caused by diseases such as human oral cancer will be necessary to strengthen the possibility that CCL28 assists in diagnosing human diseases.

## 5. Conclusions

A constant supply of saliva is required in the oral cavity to maintain a proper oral environment and protect the host’s health. Therefore, developing approaches to evaluate the functionality of salivary glands is highly beneficial for promoting human health.

In the present study, we demonstrated using CCL28-deficient mice that CCL28 maintained functional SMG stem cells, promoted the structural organization of the SMG, and regulated the production of mucus saliva, which helps protect against bacterial invasion. The effects of CCL28 on salivary gland tissue structure and function are essential activities deeply related to the saliva production ability of the salivary glands. In addition, these are unique characteristics of CCL28 that were previously unknown. Consequently, CCL28 could be responsible for maintaining the oral environment and effectively improving oral and general health, thereby contributing to human health.

## Data Availability

The data that support the findings of this study are available from the corresponding author upon reasonable request.

## Supplementary Materials

The following supporting information can be downloaded at: https://www.mdpi.com/article/10.3390/biology13030147/s1, Figure S1. Gram staining of SLG in WT and CCL28-deficient mice; Figure S2. CCL28 and CCR10 expression in the SMG.
