# Supplementary material for: CCL28: A Promising Biomarker for Assessing Salivary Gland Functionality and Maintaining Healthy Oral Environments
Source: Biology (Basel). 2024 Feb 27;13(3):147. doi: 10.3390/biology13030147 (PMC10968457; doi:10.3390/biology13030147)
Supplement: Supplementary file 1 [file biology-13-00147-s001.zip › Fig.S1.pdf]

Figure S1

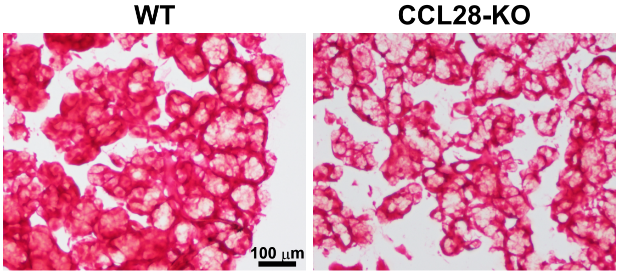

Figure S1. Gram staining of SLG in WT and CCL28-deficient mice.

Sections of the SLG from WT or CCL28-KO mice were stained with Gram-Hucker's solution. Representative images are shown. Scale bar: 100 μm.
