# Supplementary material for: CCL28: A Promising Biomarker for Assessing Salivary Gland Functionality and Maintaining Healthy Oral Environments
Source: Biology (Basel). 2024 Feb 27;13(3):147. doi: 10.3390/biology13030147 (PMC10968457; doi:10.3390/biology13030147)
Supplement: Supplementary file 1 [file biology-13-00147-s001.zip › Fig.S2.pdf]

Figure S2

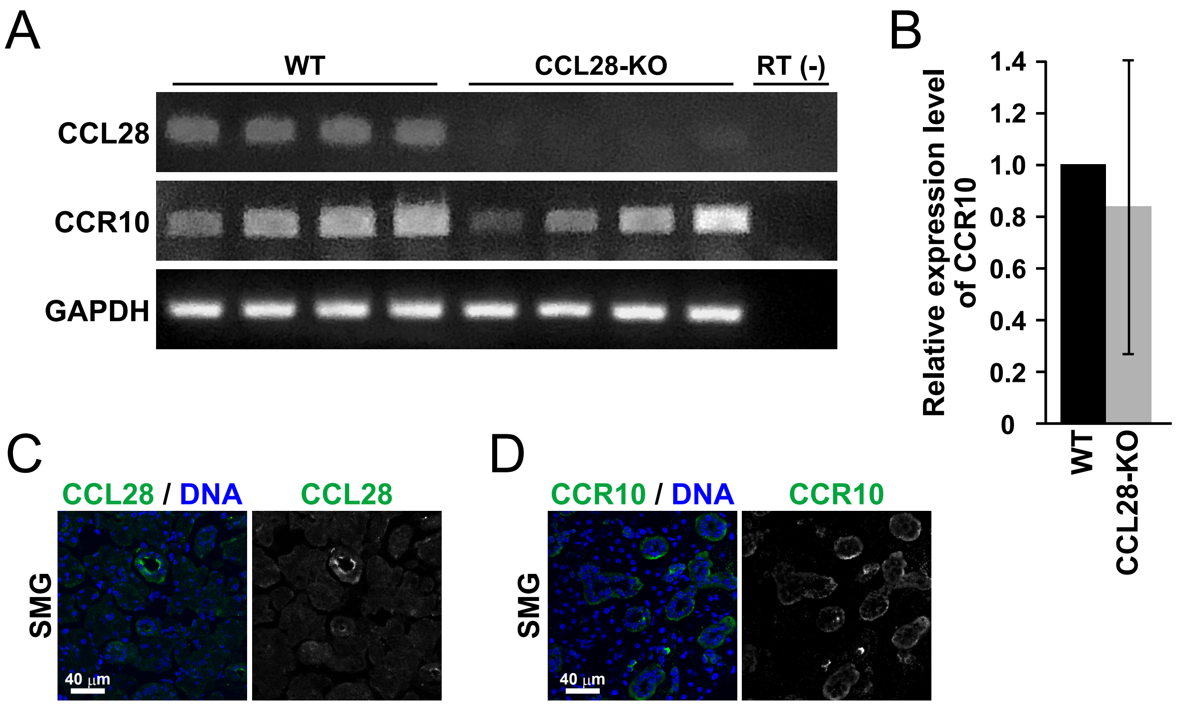

Figure S2. CCL28 and CCR10 expression in the SMG.

(A) Semi-quantitative RT-PCR for mRNA expression of CCL28, CCR10, and GAPDH was performed using cDNA prepared from the SMG derived from each of the four WT or CCL28-KO mice.

(B) The relative expression level of the CCR10, analyzed using the values obtained by measuring the bands in (A), is shown as the means ± SD of results from four independent mice.

(C, D) Sections of the SMG from WT mice were stained for CCL28 (C) (green in the left panel) or CCR10 (D) (green in the left panel) and DNA (blue in the left panel). Representative images are shown. Scale bar: 40 μm. The right panel shows the same image as the left panel, with the blue signal of the nuclear stain omitted and only the green signals of CCL28 or CCR10 shown in white.
